# Correlation between cognition and plasma noradrenaline level in Alzheimer’s disease: a potential new blood marker of disease evolution

**DOI:** 10.1038/s41398-020-0841-7

**Published:** 2020-07-03

**Authors:** Laure-Elise Pillet, Camille Taccola, Justine Cotoni, Hervé Thiriez, Karine André, Romain Verpillot

**Affiliations:** 1 Alzohis, 28 Rue du Faubourg Poissonnière, 75010 Paris, France; 2 Statitec, Groupe MultiHealth, Vélizy Espace—Immeuble Santos Dumont, 13 Avenue Morane Saulnier, 78140 Vélizy, Villacoublay France

**Keywords:** Physiology, Molecular neuroscience

## Abstract

Recent evidence showing degeneration of the noradrenergic system in the locus coeruleus (LC) in Alzheimer’s disease (AD) has motivated great interest in noradrenaline (NA) as a potential brain hallmark of the disease. Despite the current exploration of blood markers for AD, the deregulation of the plasma NA concentration ([NA]_plasma_) in AD is currently not well understood. This retrospective study includes a cohort of 71 patients (32 AD patients, 22 with other dementia and 17 without dementia) who were given consultations for memory complaints in the Cognitive Neurology Center of Lariboisière (Paris) between 2009 and 2014. As previously described in brain tissue, we show for the first time a linear correlation between [NA]_plasma_ and Mini Mental State Examination (MMSE) score in AD patients. We observed that high [NA]_plasma_ in AD patients was associated with higher [Aβ_1–42_]_CSF_ than in other AD patients with [NA]_plasma_ similar to NC patients. In parallel, we observed a lower (p-Tau/Tau)_CSF_ in AD patients with low [NA]_plasma_ than in non-AD patients with [NA]_plasma_ similar to [NA]_plasma_ in NC patients. Our data suggest that [NA]_plasma_ could be a potential biomarker of disease evolution in the context of AD and could possibly improve early diagnosis.

## Introduction

Alzheimer’s disease (AD) is the most common cause of dementia among adults over 65 years old and is characterized by progressive impairment in cognitive function and behavior^1^. The neuropathological hallmarks of the disease include amyloid plaques, formed by extracellular accumulation of amyloid β peptide (Aβ_1–42_) and neurofibrillary tangles, formed by intraneuronal aggregates of phosphorylated Tau protein p-181 (p-Tau)^2^. Cerebrospinal fluid (CSF) core biomarkers (Aβ_1–42_, p-Tau, and total-Tau), as well as amyloid and Tau positron-emission tomography, reliably reflect AD neuropathological brain lesions^3,4^, leading to their inclusion in research diagnostic criteria. However, acquiring these measurements is costly and invasive. In addition, CSF biomarkers are sometimes not sufficient to differentiate AD from other types of dementia, such as dementia with Lewy bodies^5^. Indeed, differential diagnosis remains a major challenge in clinical practice^6^. Thus, addressing the need to develop novel and inexpensive diagnostic techniques has become a focal point of Alzheimer’s research^2^.

## The noradrenergic system and AD

Many studies have shown that the abnormal structure and function of noradrenergic neurons are closely related to AD pathophysiology^6–8^. The noradrenergic system plays pivotal regulatory roles in various behaviors, including selective attention, memory storage and retrieval, vigilance, and mood^9^. Most of the noradrenergic neurons in the CNS originate from the locus coeruleus (LC) and project to different areas of the brain, such as the cortex, hippocampus, amygdala, thalamus, and hypothalamus^10^. Hyperphosphorylation of Tau protein in the LC appears at an early stage of AD pathogenesis^11^, and the number of neurons in the LC has been shown to progressively decrease during the disease, beginning at the prodromal stage of AD^8,12–17^. Based on the current literature, one of the ideas that has emerged is that the onset of AD is preceded by abnormal hyperactivation of the LC, resulting in oversecretion of NA in the cortex^18^. The hyperactivation of noradrenergic receptors in the cortex leads to a cortical accumulation of Aβ_1–42_ plaques^19^. Following this accumulation, the dendritic spine density of noradrenergic neurons and noradrenaline (NA) secretion begin to decrease due to lack of stimulation and/or (glial) inflammatory processes^18^. However, this working hypothesis requires further research in this area and supporting evidence. NA is a catecholamine that acts as a neuromodulator when synthesized by noradrenergic neurons and as a hormone when synthesized by the sympathetic system. NA deregulation in AD was first identified at the end of the 1970s by Adolfsson et al., who showed that the NA concentration in postmortem brain tissue was lower in AD patients of ~75 years of age than in age-matched controls and correlated with dementia score^20^. Interestingly, a recent publication reported a correlation between the NA concentration in a specific cortical brain region of AD patients and MMSE score^21^. In addition, NA CSF levels in advanced AD patients differ from those in patients with mild to moderate severity and those in healthy older subjects^22,23^. These observations imply that cognitive decline could be timely correlated with coeruleo-cortical network deregulation and disconnection^18^. The early increase in neuronal NA secretion before the onset of AD that is later followed by noradrenergic neuron degeneration could be related to disease evolution^11,24^. At the peripheral level, NA circulates through the bloodstream and is known to be involved in the body response to acute stress by modulating, among other physiological parameters, heart rate, blood pressure, and muscle contraction^25^. Interestingly, the LC is known to regulate sympathetic activity^26^ by modulating postsympathetic noradrenergic neurons targeting the heart and blood vessels^27^. Different studies have shown that the plasma NA concentration ([NA]_plasma_) is altered in AD. Although these studies show conflicting results, it seems clear that AD pathophysiology is associated with modulations in [NA]_plasma_^23,28–30^.

## Early stage of AD: CSF and plasma biomarkers

Investigations of potential treatments for AD have revealed the need to define and characterize an early stage of the disease, where therapeutic treatments could be the most effective. Recent studies have attempted to identify new biomarkers that can identify AD patients before the onset of advanced symptoms. The term mild cognitive impairment (MCI) was used for the first time in the 1980s to define patients at an intermediate stage between normal aging patients and patients with dementia^31^. Recently, the National Institute on Aging and Alzheimer’s Association (NIA-AA) specified the state “MCI due to AD” to clearly identify a very early stage of this disease^32^. Among other clinical criteria^33^, CSF biomarkers are required in the determination of MCI due to AD, such as CSF Aβ_1–42_, total-Tau, and/or p-Tau concentrations^32^. Several studies in the literature have shown a significant difference in CSF Aβ_1–42_^34–36^, total-Tau^35–39^, and p-Tau^37^ concentrations between MCI and AD patients, describing a decrease in the CSF Aβ_1–42_ concentration ([Aβ_1–42_]_CSF_) and an increase in the CSF total-Tau and p-Tau concentrations ([Tau]_CSF_ and [p-Tau]_CSF_) in AD patients. However, these differences are not always statistically significant for CSF Aβ_1–42_^37,40^, total-Tau^34,40^, and p-Tau^34,35,39,40^. A higher CSF p-Tau/total Tau ratio ((p-Tau/Tau)_CSF_) (or lower (Tau/p-Tau)_CSF_) was also previously described in AD patients in comparison with healthy controls^41,42^. Furthermore, (p-Tau/Tau)_CSF_ has been shown to be lower in AD patients than in control patients and patients with fronto-temporal dementia^43^. However, no difference in (p-Tau/Tau)_CSF_ was found between patients with mild AD and those with moderate AD^41^. (p-Tau/Tau)_CSF_ was also associated with the rate of cognitive decline, showing a negative dose-dependent relation between (p-Tau/Tau)_CSF_ and yearly MMSE score fold change during AD^44^. In other words, a low (p-Tau/Tau)_CSF_ in patients with AD is associated with a faster cognitive decline than that observed in other AD patients with higher (p-Tau/Tau)_CSF_. This association implies that AD patients with low [Aβ_1–42_]_CSF_ and elevated [Tau]_CSF_ and [p-Tau]_CSF_ are more likely to be in an advanced stage of the disease.

Although these results are conflicting, the CSF AD biomarker profile seems helpful in the differentiation of MCI patients from AD patients and provides information about whether the patients is in an early or late stage of the disease.

The need to find alternative body fluids for biomarker identification in AD has recently led investigators to analyze currently known CSF biomarkers for AD in plasma^45–57^. Studies in this area have shown conflicting results with (i) no difference between AD and control patients^47,49,50^, (ii) differences in plasma Aβ_1–42_^45,58^, Tau^57^ and p-Tau^54^ concentrations between AD and control patients, and (iii) differences in plasma Aβ_1–42_^53,58^, Tau, and p-Tau^54^ levels among patients with MCI due to AD, patients with AD, and control subjects. Other articles have reported that the plasma Aβ_1–42_ concentration might change during disease evolution, showing abnormally high concentrations at the preclinical stage or beginning of cognitive decline that decrease progressively during AD progression^51,55^. Moreover, other studies have shown different linear correlations in the context of AD (i) between imaging data of amyloid plaque deposition in the brain and CSF^59^ or plasma^46,48^ biomarker concentrations and (ii) between CSF and plasma biomarker concentrations^45,47,50,57,58^. The mechanisms behind brain AD biomarker clearance pathways (blood–brain, CSF–brain and blood–CSF barriers) are not yet understood, and it is not clear if those barriers are altered during the disease. Nonetheless, some articles suggest that biomarkers in plasma could mirror brain and CSF biomarker deposition and metabolism^60^. However, some studies did not observe any correlation between CSF and plasma biomarker concentrations^56^. There are a variety of possible factors that could explain the inconsistent conclusions and concentration value heterogeneity among studies. For example, technical (antibody used, time and temperature of sample conservation, hydrophobic and albumin binding properties of Aβ, low concentration in comparison with CSF, etc.) and physiological (effect of age and diet, peripheral origins, kidney and hepatic clearance, etc.) reasons^51,58^ may make determining plasma Aβ_1–42_ level alterations in the context of AD difficult. For these reasons, the clinical utility of plasmatic Aβ_1–42_ and Tau has not yet been demonstrated, and the need to identify other plasma AD biomarkers is crucial for AD diagnosis.

In this retrospective study, we explored [NA]_plasma_ in patients between 58 and 79 years old consulting for memory complaints for the first time. Our study examined the relationship between [NA]_plasma_ and concomitant diagnostic criteria such as MMSE score and CSF biomarker profile (Aβ_1–42_, Tau and p-Tau). Due to the deregulation of noradrenergic transmission in the brain during AD, we wanted to determine whether [NA]_plasma_ could be correlated with clinical parameters reflecting the stage of the disease at the cognitive (MMSE score) and molecular (Aβ_1–42_, Tau, and p-Tau CSF biomarkers) levels.

## Materials/subjects and methods

### Study population

All patients presented to the Cognitive Neurology Center of Lariboisière (Paris) for their first consultation between 2009 and 2014. Patients involved in this study were between 58 and 79 years old at the time of blood sampling. MMSE score and lumbar puncture were performed the day of blood sampling or <1 month later. The MMSE is a worldwide commonly used, easy to apply and rapid screening tool lasting <10 min. The MMSE score provides supporting information for dementia diagnosis that evaluates global cognitive impairment for all causes of dementia^61^. Briefly, the paper-based test consists of 11 orally administered questions with verbal and written responses concerning different domains (attention, memory, orientation, language, and ability to follow verbal and written commands) with a maximum score of 30 (normal cognition) and lower scores highlighting a severe deficit^62^. The original article published in 1975 defined 24 as a cutoff score for normal cognition with a sensitivity and specificity of 87% and 82.6%, respectively^61^. In a more recent study, the mean MMSE score of the population older than 90 years without dementia was reported to be 26.6, whereas the cutoff score for normal cognition was reported to be 23.3, very similar to the cutoff score for normal cognition in the younger population (~23 or 24 points)^62,63^. In the context of AD, the MMSE score is used to assess disease severity and is occasionally used to estimate efficacy in clinical drug trials^64^. Previous studies have shown that MMSE scores decrease during AD progression at different rates depending on disease progression and patient education^65^. More generally, patients in the moderate to severe stages of AD have an MMSE score below 20^66^. Sample size was calculated based on results from previous articles highlighting a significant difference in plasma catecholamines between AD and non-AD patients. Raskind et al.^23^ observed a significant difference between advanced AD and control patients with a small sample size (<20 patients). Therefore, we based our calculation for sample size on the article of Umageki et al.^28^ that examined adrenaline levels. Considering a common standard deviation of 18.46 pg/mL, a sample size of 68 patients is necessary to show a difference of 12.69 pg/mL between the two groups with a power of 80% and an α of 5%. Seventy-one patients were included in this retrospective study**:** 32 AD patients (diagnoses were performed according to NIA-AA guidelines^4^), 22 other dementia (OD; frontotemporal dementia, vascular dementia, or dementia with Lewy bodies) patients, and 17 neurological control (NC) patients. NC patients were defined as those with memory complaints, mental depression or anxiety but for whom no dementia was diagnosed. The following cutoff values for core AD CSF biomarkers were used as supportive criteria for dementia due to AD: Aβ1–42 (<550 pg/mL), total-Tau (>400 pg/mL), and p-Tau (>50 pg/mL). Demographic information, MMSE scores, presence of the APOE ε4 allele, CSF biomarkers (Aβ_1–42_ protein, total Tau, and p-Tau protein) and concomitant medications of the studied cohort are shown in Table 1.

**Table 1:** Demographic and physiologic data of studied cohort.

		NC	OD	AD	*p* value
**Total number of patients**		17	22	32	–
Sex	% of female patients	52.9	36.4	56.3	0.3360
Age	Age mean (SD) in year	67 (6.671)	67.27 (5.650)	70.69 (6.703)	0.0746
MMSE	MMSE score median (IQR)	28 (27–29)	21 (16.25–23.25)	23 (20–25)	<0.0001
*APOE4*^a^	% of patients carrying *APOE* ε4 allele	15.4	36.8	70.0	0.0021
CSF Aβ_1–42_ concentration^b^	CSF Aβ concentration mean (SD)	795.4 (155,8)	793.1 (294.5)	419.2 (162.2)	<0.0001
CSF Tau concentration^b^	CSF Tau concentration median (IQR)	172 (139–239.5)	245 (190–299)	581.5 (388.8–766.8)	<0.0001
CSF p-Tau concentration^b^	CSF p-Tau concentration median (IQR)	34.50 (19.50–44.75)	44 (34–59.50)	88.5 (69.45–113.50)	<0.0001
Plasma NA concentration	Plasma NA concentration median (IQR)	2564 (1614–3131)	2108 (1540–2561)	2194 (1846–3534)	0.3873
% of patients with co-medication	Anti-Alzheimer or anti-Parkinsonian/dopaminergic agents	5.9	22.7	18.8	0.3536
	Antidepressants	23.5	27.3	21.9	0.9000
	Benzodiazepines (anxiolytics/hypnotics) and Neuroleptics	5.9	18.2	9.4	0.4361
	Lipid-lowering agents, oral antidiabetics	35.3	40.9	28.1	0.6143
	Anti-hypertensive agents	52.9	31.8	31.3	0.2770
	Veinotonics / vasodilatators	0.0	0.0	0.0	–
	Others (Vitamines, anti-asthmatics, non steroidal anti-inflammatory agents)	23.5	18.2	12.5	0.6069

^a^Five NC, three OD, and two AD patients did not undergo APOE genotyping.

^b^Three NC, one OD, and two AD patients did not undergo lumbar puncture.

### Plasma NA quantification

Patients fasted overnight (for ~12 h) before blood collection and were in the decubitus position during sampling. Plasma samples were purified and analyzed with a reagent kit for HPLC analysis of catecholamines in plasma (Chromsystems, order #5000) according to the manufacturer’s instructions. Briefly, blood samples were stabilized with glutathione, and plasma was isolated less than 1 h after blood sampling by centrifugation. Plasma samples were stored at −80 °C. After thawing, 1 mL of plasma was used to extract catecholamines for dosage by high-performance liquid chromatography coupled with electrochemical detection. Experimenters did not know the corresponding group of the sample during dosage.

### CSF biomarker quantification

Lumbar punctures were performed on fasting patients, typically between 9 and 12 a.m. CSF samples were centrifuged at 1 *g* for 10 min at 4 °C within 4 h of collection, aliquoted in 0.5-mL polypropylene tubes and stored at –80 °C for further analysis. CSF levels of Aβ_1–42_, total Tau, and p-Tau were measured using the commercially available sandwich ELISA INNOTEST^®^, according to the manufacturer’s procedures (Fujirebio Europe NV, formerly Innogenetics NV).

### Statistical analysis

Depending on the normality of the data (D’Agostino-Pearson normality test), the results are presented as the mean with standard deviation (SD) (standard error of mean in figures) or median with interquartile range (IQR: 25–75th percentiles) (95% confidence interval in figures). For normally distributed data, we performed Student’s *t* test (or Student’s *t* test with Welch’s correction if the *F*-test showed significantly different variances between groups) or one-way ANOVA. For non-normally distributed samples, we performed a Mann–Whitney test or Kruskal–Wallis test. Fisher’s exact test was used to compare frequencies. Linear correlation coefficients were calculated using Pearson’s correlation test or Spearman’s correlation test for normally or not normally distributed data, respectively. To compare distributions of data, we performed the Kolmogorov–Smirnov test. We performed Rosner’s Extreme Studentized Deviate test (using log-normal distribution for plasma and CSF biomolecules) for multiple outliers (two-sided test) with a *p* value of 0.01, and we found no outliers for MMSE score, [NA]_plasma_, [Aβ_1–42_]_CSF_, [Tau]_CSF_, or [p-Tau]_CSF_. Analyses were performed using GraphPad Prism 8.0.1 software. Statistical significance was set at *p* value < 0.05.

## Results

### Characterization of the study cohort

The studied groups did not significantly differ by sex ratio, age, or concomitant treatments (Table 1). As expected, they differed by MMSE score and by *APOE* ε4 carrier status (Table 1). Clinical diagnosis of AD made by the neurologist was based on age, MMSE score, and CSF biomarkers, according to NIA-AA guidelines^4^. AD patients had significantly lower Aβ_1–42_, higher p-Tau, and higher total-Tau CSF concentrations than OD and NC patients (Table 1).

### Correlation between plasma NA concentration and cognitive MMSE score in AD patients

**Fig. 1:**
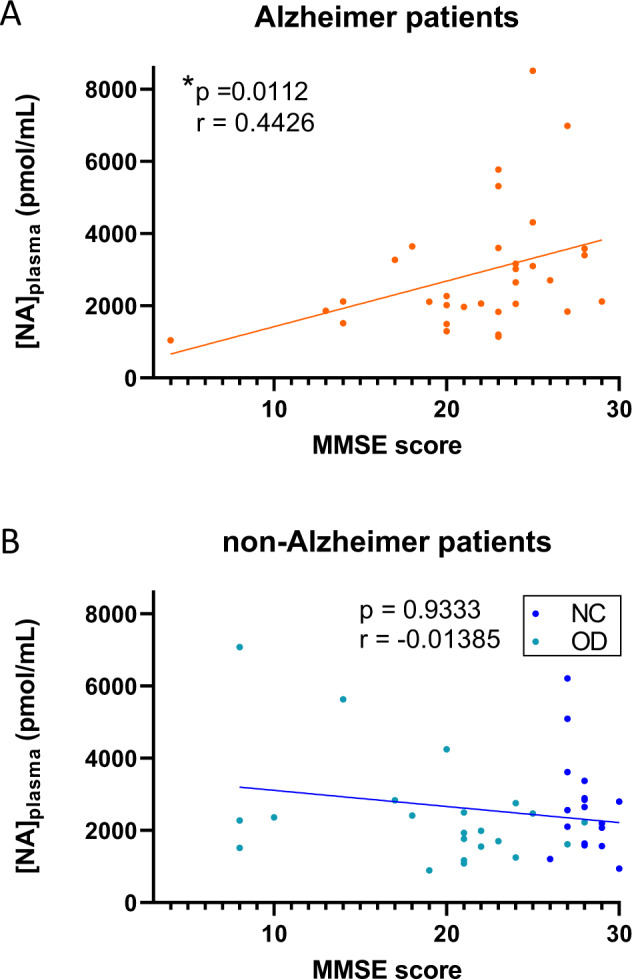
MMSE score correlates with [NA]_plasma_ in AD patients. Linear regression between [NA]_plasma_ and MMSE score of AD (**a**) and non-AD (**b**) patients. * indicates *p* value < 0.05.

**Fig. 2:**
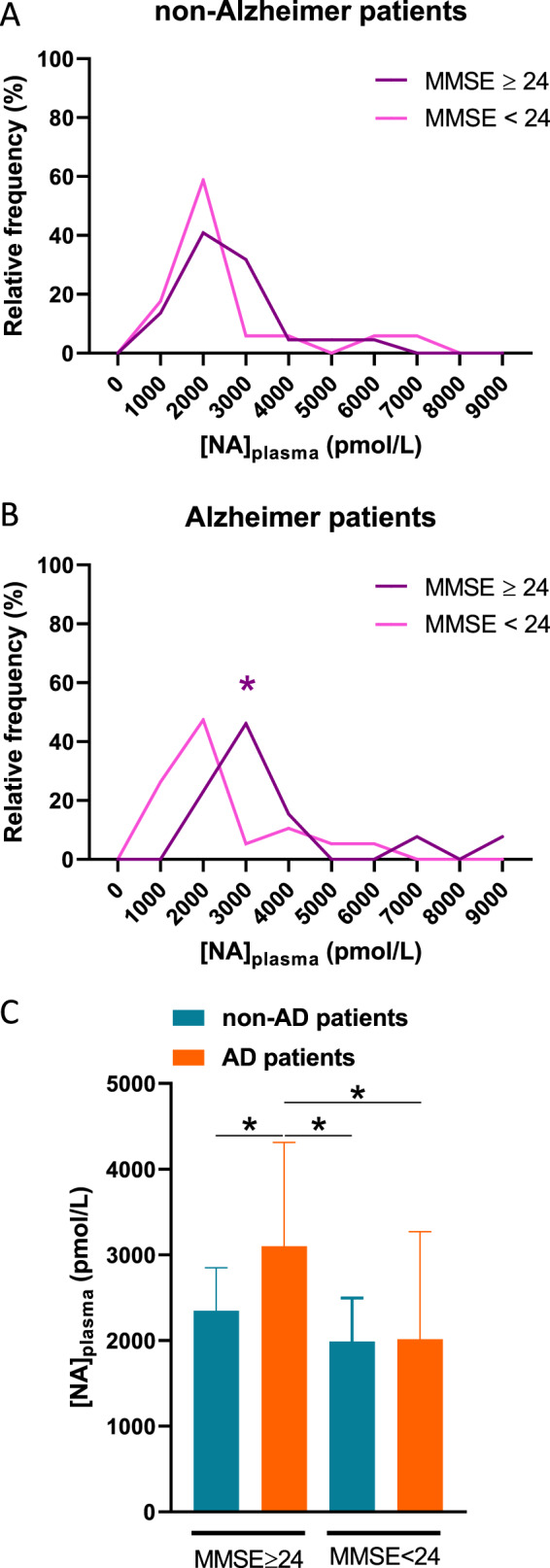
Existence of subpopulations of AD patients described by MMSE score and [NA]_plasma_. **a** Relative frequency distribution of [NA]_plasma_ in non-AD patients with MMSE score above and below 24. **b** Relative frequency distribution of [NA]_plasma_ in AD patients with MMSE score above and below 24. **c** [NA]_plasma_ median of NC, OD, and AD patients with MMSE score above and below 24. * indicates *p* value < 0.05.

As previously described in a specific cortical brain region^21^, we observed a significant linear correlation between [NA]_plasma_ at the peripheral level and MMSE score in AD patients (Spearman’s correlation, *r* = 0.4426 (95% CI: 0.1004–0.6912); *p* value = 0.0112; equation: *Y* = 126.3*X + 158) (Fig. 1a). However, this correlation was not observed in non-AD patients (Spearman’s correlation, *r* = −0.01385 (95% CI: −0.3365 to 0.3117); *p* value=0.9333; equation: *Y* = −44.63*X + 3556) (Fig. 1b). These results suggest a possible link between [NA]_plasma_ and cognitive decline in AD patients. Nonetheless, we observed no difference in [NA]_plasma_ among the AD, OD and NC patients (Table 1). We discriminated patients with an MMSE score above (≥) and below (<) 24, which correspond to the cutoff score for normal cognition in the literature^67^. In non-AD patients, we observed no difference in the distribution of [NA]_plasma_ between patients with an MMSE score above and those with a score below 24 (Kolmogorov–Smirnov test, *p* value = 0.7459) (Fig. 2a). On the other hand, we found a significant difference between the distribution of [NA]_plasma_ in AD patients with an MMSE score above and those with a score below 24 (Kolmogorov–Smirnov test, *p* value = 0.0260) (Fig. 2b). Moreover, we observed that the median [NA]_plasma_ of AD patients with an MMSE score above 24 was significantly higher than the median [NA]_plasma_ of non-AD patients with a similar MMSE score (≥24) (Mann–Whitney test, *p* value = 0.0287) and lower (<24) MMSE score (Mann–Whitney test, *p* value = 0.0136) and than the median [NA]_plasma_ of other AD patients (Mann–Whitney test, *p* value = 0.0177). We observed no difference between the median [NA]_plasma_ of AD patients with an MMSE score below 24 and that of non-AD patients with similar (Mann–Whitney test, *p* value = 0.8757) or higher (Mann–Whitney test, *p* value = 0.3851) MMSE scores (Fig. 2c). Taken together, these results support the idea that there is a subpopulation of AD patients with an MMSE score above 24 and a higher median {NA]_plasma_ than other patient groups, implying that a mild cognitive decline in AD depicted by MMSE score is associated with an elevated [NA]_plasma_.

### NA plasma level in AD is associated with MMSE score and CSF biomarker profile

**Fig. 3:**
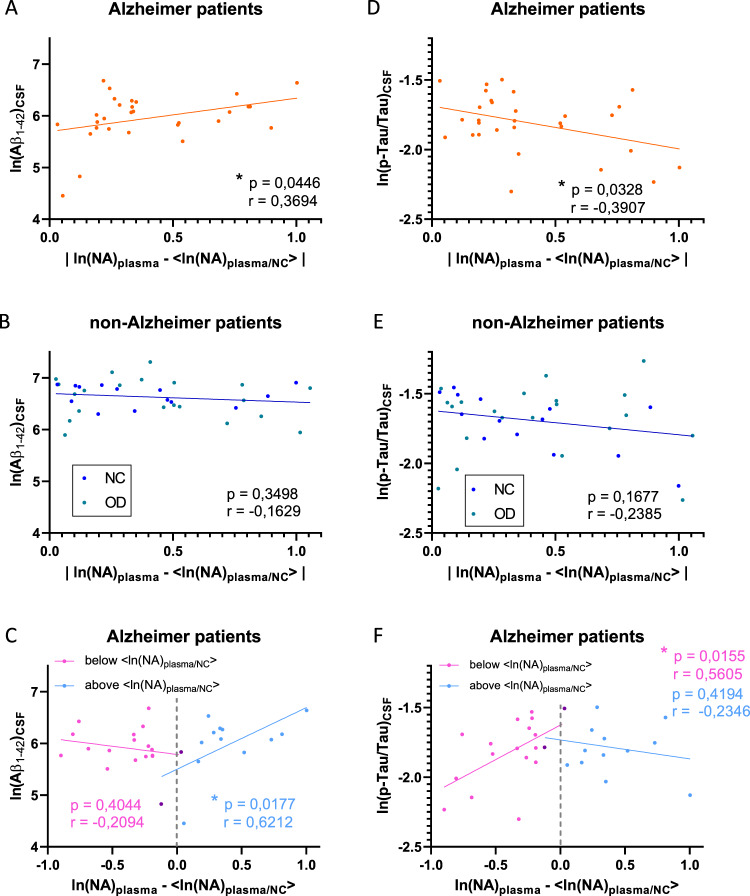
Absolute [NA]_plasma_ distance from median of NC patients correlates with [Aβ1–42]_CSF_ and (p-Tau/Tau)_CSF_ in AD patients. Linear correlations between absolute distance of ln(NA)_plasma_ from median ln(NA)_plasma/NC_ with [Aβ_1–42_]_CSF_ in AD (**a**) and non-AD (**b**) patients. **c** Linear correlations between negative (in pink) and positive (in blue) values of ln(NA)_plasma_-<ln(NA)_plasma/NC_>with [Aβ_1–42_]_CSF_ in AD patients (points in purple were counted in both groups). Linear correlations between absolute distance of ln(NA)_plasma_ from median ln(NA)_plasma/NC_ with (p-Tau/Tau)_CSF_ in AD (**d**) and non-AD (**e**) patients. **f** Linear correlations between negative (in pink) and positive (in blue) values of ln(NA)_plasma_-<ln(NA)_plasma/NC_>with (p-Tau/Tau)_CSF_ in AD patients (points in purple were counted in both groups). * indicates *p* value < 0.05.

To determine whether [NA]_plasma_ reflects early disease evolution, we examined whether there was a correlation between the distance from the NC patient median [NA]_plasma_ and the CSF biomarker profile. Because of the non-normal distribution of [NA]_plasma_ data, we decided to use logarithmic data as previously described^68^ (in the following text, all statistical tests for CSF biomarkers were performed on logarithmic data). This distance was defined as the absolute value of ln(NA)_plasma_—<ln(NA)_plasma/N_>, with <ln(NA)_plasma/N_> the median value of logarithmic [NA]_plasma_ from NC patients (<ln(NA)_plasma/NC_ ≥ 7.849). We found a significant positive linear correlation between this absolute distance and [Aβ_1–42_]_CSF_ in AD patients (Pearson’s correlation, *r* = 0.3694 (95% CI 0.01047 to 0.6439); *p* value = 0.0446; equation: *Y* = 0.6449*X + 5.695) (Fig. 3a), but this correlation was not observed in non-AD patients (Pearson’s correlation, *r* = −0.1629 (95% CI −0.4706 to 0.1801); *p* value = 0.3498; equation: *Y* = −0.1662*X + 6.697) (Fig. 3b). Looking at the relative distance of ln[NA]_plasma_ from <ln(NA)_plasma/NC_ > (negative or positive when respectively lower or higher than the NC patients median), we observed a negative correlation that does not reach significance for negative distance (Pearson’s correlation, *r* = −0.2094 (95% CI −0.6160 to 0.2854); *p* value = 0.4044; equation: *Y* = −0.3175*X + 5.786) and a significant positive correlation for positive distance (Pearson’s correlation, *r* = 0.6212 (95% CI: 0.1352–0.8663); *p* value = 0.0177; equation: *Y* = 1.192*X + 5.499) (Fig. 3c). Similarly, we found a negative linear correlation between absolute distance from <ln(NA)_plasma/N_> and (p-Tau/Tau)_CSF_ in AD patients (Pearson’s correlation, *r* = −0.3907 (95% CI: −0.6583 to −0.03541); *p* value = 0.0328; equation: *Y* = −0.3079*X – 1.687) (Fig. 3d) but not in non-AD patients (Pearson’s correlation, *r* = −0.2385 (95% CI: −0.5296 to 0.1029); *p* value = 0.1677; equation: *Y* = −0.1737*X – 1.621) (Fig. 3e). An examination of the relative distance from <ln(NA)_plasma/NC_> revealed a significant positive correlation for negative distance (Pearson’s correlation, *r* = 0.5605 (95% CI: 0.1269–0.8143); *p* value = 0.0155; equation: *Y* = 0.4962*X – 1.625) and a negative correlation for positive distance that does not reach significance (Pearson’s correlation, *r* = −0.2346 (95% CI: −0.6805 to 0.3380); *p* value = 0.4194; equation: *Y* = −0.1361*X – 1.732) (Fig. 3f). Taken together, our results suggest that [NA]_plasma_ may reflect the progression of AD, highlighting the existence of different groups of AD patients characterized by different biomarkers.

## Discussion

There is a significant amount of literature on deregulation of cerebral, plasma, and urine NA concentrations in murine models of AD^69–72^ and in human AD patients^21,28,29,73,74^. In mouse models, NA has been implicated in the microglial phagocytosis of Aβ_1–42_ plaques and cognition^24,75^. In humans, NA levels in cortical brain regions have been correlated with dementia score^20^ and MMSE score^21^, which decreases during AD development^67^. This observation is consistent with our results showing a significant correlation between [NA]_plasma_ and MMSE score in AD patients. Regarding those results, it is tempting to say that [NA]_plasma_ could be a mirror of cerebral NA deregulation and could help in the diagnosis of AD. Previous studies have shown an increase^23^ or a decrease^28^ in [NA]_plasma_ in AD patients in comparison with the concentration in control patients. This discordance could be explained by the existence of subpopulations of AD patients, as suggested previously^23^ and as identified in our analysis as patients with a high distance from the median [NA]_plasma_ of NC patients (meaning high or low [NA]_plasma_).

In the present study, high [NA]_plasma_ in AD patients was associated with higher [Aβ_1–42_]_CSF_ than in other AD patients with [NA]_plasma_ similar to NC patients. Several studies in the literature have compared [Aβ_1–42_]_CSF_ between MCI stage and more or less severe AD. MCI patients have been shown to have a higher [Aβ_1–42_]_CSF_ than AD patients^34–37,40^. There have also been conflicting results concerning CSF Tau and p-Tau concentrations, showing no difference or increased levels in late-stage AD compared with MCI^34–37,40,41,76^. However, the differences observed were not always significant. The absence of statistical significance could be due to several parameters that differ among the different studies, such as the precision of the chemical dosage, sample storage, age of patients, sex ratio of the cohort groups, MMSE score, or advancement of the disease, involving stages of AD more or less distant from MCI. Moreover, the temporal evolution of biomarkers and cognitive markers of AD have previously been shown to differ according to disease stage (asymptomatic, MCI and dementia stage)^77^. We observed a lower (p-Tau/Tau)_CSF_ in AD patients with low [NA]_plasma_ than in non-AD patients with [NA]_plasma_ similar to [NA]_plasma_ in NC patients. A low (p-Tau/Tau)_CSF_ has also been associated with faster cognitive decline^44^, suggesting a possible link between this ratio and disease severity. In this way, low (p-Tau/Tau)_CSF_ associated with low MMSE score and low [Aβ_1–42_]_CSF_ is more likely to describe an advanced stage of AD pathology.

Regarding MMSE score, [Aβ_1–42_]_CSF_, and (p-Tau/Tau)_CSF_, it is tempting to speculate that AD patients with high or low [NA]_plasma_ represent an early or late stage of AD, setting [NA]_plasma_ as a potential marker of disease evolution. However, the link between the brain and [NA]_plasma_ alterations during AD is not yet understood. We hypothesize that the [NA]_plasma_ increase observed in AD patients could be due to (i) NA oversecretion in the CNS and disruption of the blood-brain barrier that may occur in AD^78^, (ii) release of cerebral NA in the blood through the CSF interface, with the potential of a linear correlation between [NA]_CSF_ and [NA]_plasma_^23^, (iii) deregulation of NA secretion into the blood by the sympathetic system regulated by the LC^26^, and (iv) NA oversecretion from sympathetic noradrenergic postganglionic neurons to targeted organs and leakage or spillover into the blood^79^. We did not approach the concept of cognitive reserve in this article. Cognitive reserve is the phenomenon that maintains cognitive function despite advanced physiological symptoms of AD pathology in the brain^80^. Robertson developed the idea that LC-noradrenergic system activity may be the biological mechanism explaining the gap between cognitive function and pathophysiological observations of the disease (especially amyloid plaques)^81^, which has been further supported by several articles^82–84^. The pupillary response, a potential marker of NA-LC activity, illustrates that NA activity is enhanced by cognitively challenging stimuli. Knowing that NA seems to mediate neurocompensatory and neuroprotective effects, variables of cognitive reserve (education, enriched environment/novelty, social interactions, mental activity) may lessen AD pathology through noradrenergic pathway activation^81^. Thus, this pathway represents a good candidate for the missing link in the cognition-brain pathology discrepancy observed in AD. Robertson has highlighted the fact that cognitive reserve phenomena through the action of NA in AD could have a direct effect on AD symptoms (anti-inflammatory action, inhibition of amyloid aggregation, etc.) and/or a compensatory effect (increased connectivity, network reorganization). This complex question remains open and needs to be explored with further studies.

In this study, we showed for the first time that [NA]_plasma_ was correlated with MMSE score in the context of AD. We described particular profiles of MMSE score and CSF AD biomarkers for AD patients with high and low [NA]_plasma_. [NA]_plasma_ deregulation could hypothetically reflect overactivation and regression of the LC during AD. These results imply that (1) [NA]_plasma_ could support the early diagnosis of AD, (2) [NA]_plasma_ could help to better characterize AD patient profiles during disease evolution, and (3) the link between LC degeneration and [NA]_plasma_ in the context of AD needs to be further investigated. Combined with the MMSE score, [NA]_plasma_ could help the patient be referred to a specialist before the appearance of advanced AD symptoms. For example, physicians could advise patients with memory complaints, good MMSE scores and high [NA]_plasma_ to consult a neurologist for further examinations. Thus, this process could favor fast and early diagnosis of AD, which opens new research potentials for blood biomarkers in AD.
