# Correction: Correlation between cognition and plasma noradrenaline level in Alzheimer’s disease: a potential new blood marker of disease evolution

**DOI:** 10.1038/s41398-020-01102-y

**Published:** 2020-11-24

**Authors:** Laure-Elise Pillet, Camille Taccola, Justine Cotoni, Hervé Thiriez, Karine André, Romain Verpillot

**Affiliations:** 1 Alzohis, 28 Rue du Faubourg Poissonnière, 75010 Paris, France; 2 Statitec, Groupe MultiHealth, Vélizy Espace—Immeuble Santos Dumont, 13 Avenue Morane Saulnier, 78140 Vélizy, Villacoublay France

**Keywords:** Physiology, Molecular neuroscience

Correction to: *Translational Psychiatry*

10.1038/s41398-020-0841-7, published online 3 July 2020

We have updated this Article since its original publication in order to change a sentence in the Acknowledgements section. Originally, the sentence relating to Alzohis and Pr. Claire Paquet read:

“We also thank Claire Paquet, MD, PhD (Memory Resources and Research Center, Cognitive Neurology Center, INSERM UMR-S 942, University Hospital of Paris Diderot Saint Louis-Lariboisière-Fernand Widal, APHP, France) for initiating and conducting this study as well as Jacques Callebert, PharmD, PhD (Department of Biochemistry, University Hospital of Paris Diderot Saint Louis-Lariboisière-Fernand Widal, APHP, France) for performing the blood sample analyses”.

We’ve changed this to:

“We also thank Claire Paquet, MD, Ph.D. (Memory Resources and Research Center, Cognitive Neurology Center, INSERM UMR-S 942, University Hospital of Paris Diderot Saint Louis-Lariboisière-Fernand Widal, APHP, France) for providing the biological and clinical samples as well as Jacques Callebert, PharmD, Ph.D. (Department of Biochemistry, University Hospital of Paris Diderot Saint Louis-Lariboisière-Fernand Widal, APHP, France) for performing the blood sample analyses”.

